# Automated surveillance of hospital-onset bacteremia and fungemia: feasibility and epidemiological results from a Dutch multicenter study

**DOI:** 10.1017/ice.2025.29

**Published:** 2025-05

**Authors:** Manon A.C.M. Brekelmans, Anne L.M. Vlek, Yvonne van Dijk, Annelies E. Smilde, Annemarie J.L. Weersink, Herman F. Wunderink, Hanneke Boon, Saara Vainio, Wendy S. Bril, Jan A.J.W. Kluytmans, Marc J.M. Bonten, Maaike S.M. van Mourik

**Affiliations:** 1 Department of Medical Microbiology and Infection Control, University Medical Centre Utrecht, Utrecht, the Netherlands; 2 Centre for Infectious Diseases Control, National Institute for Public Health and the Environment, Bilthoven, the Netherlands; 3 Department of Medical Microbiology and Immunology, Diakonessenhuis, Utrecht, the Netherlands; 4 Department of Hygiene and Infection Prevention, Diakonessenhuis, Utrecht, the Netherlands; 5 Department of Hygiene and Infection Prevention, Meander Medical Center, Amersfoort, the Netherlands; 6 Laboratory for Medical Microbiology and Medical Immunology, Meander Medical Center, Amersfoort, the Netherlands; 7 Department of Medical Microbiology and Immunology and Infection Control, St. Antonius Hospital, Nieuwegein, The Netherlands.; 8 Julius Centre for Health Sciences and Primary Care, University Medical Centre Utrecht, Utrecht, the Netherlands; 9 European Clinical Research Alliance on Infectious Diseases, Utrecht, the Netherlands

## Abstract

**Objective:**

Hospital-onset bacteremia and fungemia (HOB) has been suggested as a suitable and automatable surveillance target to include in surveillance programs, however differences in definitions across studies limit interpretation and large-scale implementation. We aimed to apply an automated surveillance system for HOB in multiple hospitals using a consensus definition, and describe HOB rates.

**Design and setting:**

Retrospective cohort study in four Dutch hospitals: 1 tertiary hospital and 3 secondary hospitals.

**Patients:**

All patients admitted for at least one overnight stay between 2017 and 2021 were included, except patients in psychiatry wards.

**Methods:**

Data from the electronic health records and laboratory information system were used to identify HOBs based on the PRAISE consensus definition. HOB rates were calculated at ward and micro-organism-level.

**Results:**

Hospital-wide HOB rates varied from 1.0 to 1.9, and ICU rates varied from of 8.2 to 12.5 episodes per 1000 patient days. The median time between admission and HOB was 8–13 days. HOBs were predominantly caused by Enterobacterales, Enterococci, *S. aureus* and coagulase-negative staphylococci. Longitudinal HOB surveillance detected differences over time at ward and micro-organism level; for example increased HOB rates were observed during the COVID-19 pandemic. Sensitivity analyses demonstrated the impact of assumptions regarding the collection of confirmatory blood cultures for common commensals.

**Conclusions:**

Applying a fully automated definition for HOB surveillance was feasible in multiple centers with different data infrastructures, and enabled detection of differences over time at ward and micro-organism-level. HOB surveillance may lead to prevention initiatives in the future.

## Introduction

Surveillance of healthcare-associated infections (HAI) aims to provide insights into the incidence of HAI to guide national and facility-level infection prevention and control (IPC) programs and reduce HAI rates.^1^ In many settings, HAI surveillance is predominantly conducted through manual chart review, a time-consuming, subjective and error-prone process.^2^

In recent years, automated surveillance (AS) has emerged to replace (parts of) manual surveillance to overcome these deficiencies. AS is defined as any form of surveillance where (parts of) the manual assessment are replaced by an automated process using routinely collected data from electronic health records (EHR).^3^ However, not all current surveillance targets are suitable for AS. In general, it is important that a surveillance target is a severe and common event that is definable and preventable.^3^ In addition, for a surveillance target to be suitable for automated surveillance, accessibility and standardization of source data are important criteria.

In the setting of large-scale implementation, a widely accepted and standardized definition that is suitable for automation is paramount and will ensure harmonization and interoperability across hospitals and surveillance networks.^4^ Furthermore, this definition needs to be applicable to different information technology systems, data infrastructures and local practices of hospitals. At the same time, the surveillance metric needs to have a sufficient level of detail to identify potential differences in infection rates and highlight areas where IPC interventions may be necessary.

In recent studies, hospital-onset bacteremia and fungemia (HOB) have been suggested as a suitable and automatable surveillance target to include in surveillance programs.^5–13^ The HOB definition is objective, includes the full spectrum of bacteremia episodes and is mainly based on microbiological data; a data source that is accessible in most centers. However, differences in definitions across studies limit interpretation and large-scale implementation. The PRAISE network recently published a detailed consensus definition for HOB that was shown to be suitable in multiple centers and multiple countries.^14^

In this study, we aimed to apply an AS system for HOB using the PRAISE definition in four Dutch hospitals in a research context and assess HOB rates at ward and micro-organism level and over time.

## Methods

### Study design and study population

This retrospective cohort study presents data collected of all hospitalized patients in four hospitals in the region of Utrecht, the Netherlands, between 2017 (Hospital 1 and 2) or 2018 (Hospital 3 and 4), and 2021. Patients admitted to psychiatry wards were excluded, as well as patients who objected to the use of their medical data for research purposes. The medical ethical review board NedMec confirmed that the Medical Research Involving Human Subjects Act (WMO) does not apply (reference number 21/856), thereby waiving the requirement for informed consent.

### Definition of hospital-onset bacteremia

**Figure 1.**
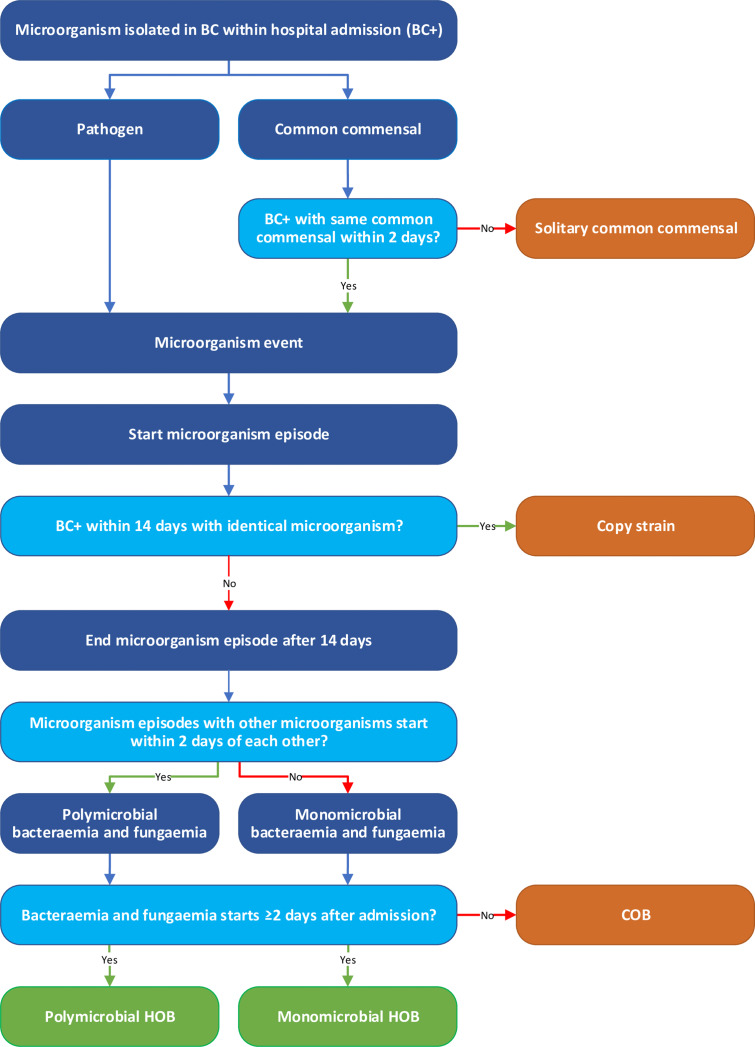
Flowchart of the algorithm identifying hospital-onset bacteremia and fungemia. *Blood cultures were defined based on set-level, ie, 1 or 2 vials. A blood culture is considered positive if a micro-organism was determined. Micro-organism events are defined by either a pathogen in 1 blood culture OR the same common commensal in 2 blood cultures (different sample ID’s) within 2 calendar days of each other. Micro-organism episodes are defined including an episode duration of 14 days. Bacteremia episodes are defined incorporating polymicrobial episodes. A bacteremia is classified as HOB if the start date is 2 or more days after hospital admission. These definitions are based on the PRAISE consensus definition, and the algorithm is described in more detail in Aghdassi et al.*^14^ *BC: blood culture, BC+: positive blood culture, HOB: hospital-onset bacteremia and fungemia, COB: community-onset bacteremia and fungemia. Figure adapted from Aghdassi et al.*^14^

The consensus definition of the PRAISE network (Providing a Roadmap for Automated Infection Surveillance in Europe) was used in our classification algorithm to identify HOBs,^14^ (Figure 1). One blood culture was defined as one set of one (pediatric vial) or two (aerobic/anaerobic vial) blood culture bottles. In case of a common commensal (NHSN classification^15^), two repeated blood cultures with the same common commensal are required to be considered as a micro-organism event. A micro-organism episode lasts 14 days, or until discharge, whatever comes first. If two episodes with different micro-organisms start within two days of each other, these were grouped in a single polymicrobial bacteremia episode. HOB was defined as a bacteremia episode starting two or more days after admission.

### Data collection

Data were extracted from the EHR and laboratory information system (LIMS) and pseudonymized within the four hospitals (data collection was performed between February and November 2022). We collected patient-, blood culture-, and admission-related data (Appendix 1, Table S1). Data sets were sent securely to the coordinating center. The data were converted into the minimal data set (MDS) format of the PRAISE consensus definition.^14^

### Data analysis

We merged data on blood cultures and admissions based on calendar dates. The first ward where the patient was located on the day of blood culture sampling was assumed to be the sampling ward. The first ward where the patient was located two days prior to blood culture sampling was the attributable ward.^14^ Hospitals classified their wards using the ECDC ward specialty classification.^16^ The midnight method was used to calculate the denominator, ie, patient days.^14^

Characteristics of all hospitals, admissions at risk (ie, admissions with a minimum length of stay of two days), admissions with at least one HOB, and all HOB episodes are presented descriptively.

The HOB incidence (ie, number of HOBs per 1000 patient days) over time was calculated by quarter as the hospital-wide HOB rate and HOB rate per ward specialty (ICU, medical wards, surgical wards and other wards). The 95% confidence interval was calculated using the Rothman-Greenland formula,^17^ or the formula of Byar when no HOB was identified in a specific group.^18^ As a time-varying reference, we calculated the mean HOB rate of the 2 years before the specific quarter. Furthermore, we depict the blood culture frequency (number of blood culture samples per 1000 patient days). HOB rates per causative micro-organism group were calculated for all hospitals together per year, distinguishing ICU and non-ICU departments.

### Sensitivity analyses

According to the PRAISE definition, common commensals are classified as a micro-organism event by the algorithm when a second positive blood culture with the same common commensal was obtained on the same day with a different sampleID and/or on day +1 or +2 after the first positive culture. In the sensitivity analysis we applied two different assumptions regarding the timing of the second positive blood culture with the same common commensal: (1) Second positive culture on the same day only, with a different sampleID (irrespective of the registered sampling time) and (2) second positive culture on day +1 or +2 after the first positive blood culture, but not on the same day. Furthermore, we explored how the number of (polymicrobial) HOBs changed when we limited polymicrobial HOB to micro-organism episodes starting on the same day, instead of within two days. Lastly, we studied the differences in results if we considered the sample ward as attributable ward.

## Results

Four hospitals were included in this study, one tertiary hospital (including a children’s hospital) and three secondary hospitals (Table 1).

**Table 1.** Baseline characteristics of the participating hospitals

	Hospital 1	Hospital 2	Hospital 3	Hospital 4
**Study period**	2017–2021	2017–2021	2018–2021	2018–2021
**Hospital type**	Tertiary	Secondary	Secondary	Secondary
**Number of admissions/year,** median (min-max)	27,349 (24,853 – 29312)	21,619 (18,737 –22,198)	41,865 (37,540 – 45,559)	23,514 (22,342 – 25,411)
**Number of patient days/year,** median (min-max)	171,578 (161,511 – 187,953)	91,257 (78,120 – 95,451)	201,519 (183,741 – 214,500)	114,268 (106,824 – 130,083)
**Range of bed numbers, n**	1000-1200	400–600	400–600	400–600
**Number of ICU beds, n**	55	12	34	15
**Length of stay (days),** median (q1-q3)	3 (1–7)	2 (1–5)	2 (1–5)	2 (1–5)
**Number of blood cultures/year,**				
Total: mean (blood culture rate/1000 patient days)	13,335 (77,7)	8,124 (89,0)	24,255 (120,4)	10,910 (95,5)
At risk: mean (blood culture rate/1000 patient days)^a^	6,940 (40.4)	2,128 (23.3)	10,820 (53.7)	3,600 (31.5)

The number of admissions and patient days were calculated for each year separately before determining the median. Number of beds includes only inpatient hospital beds. Blood culture rate is calculated as the mean number of blood cultures/median admission days * 1000. ^*a*^ *Blood cultures at risk for HOB were defined as blood cultures taken 2 or more days after admission.*

Table 2 presents baseline characteristics of admissions at risk of HOB, and admissions where at least one HOB occurred. Among the admissions at risk, 13-28% of the admissions were related to surgical specialties and 6%–30% to internal medicine (of note, only hospital 1 defines hematology/oncology as a separate admission specialty). The median length of stay was 4 or 5 days in all hospitals, with an in-hospital mortality rate ranging from 2.2% to 3.1%.

**Table 2.** Baseline characteristics for admissions at risk for HOB and with HOB

	Hospital 1		Hospital 2		Hospital 3		Hospital 4	
	at risk	with HOB	at risk	with HOB	at risk	with HOB	at risk	with HOB
**Number of admissions, n**	97,543	1365	63,976	374	99,884	1129	60,552	469
**Age**								
Age under 18 years, n (% of total)	20,200 (21)	273 (20)	7,658 (12)	4 (1)	8,615 (9)	9 (1)	5,767 (10)	6 (1)
Age under 18 years, median (q1-q3)	1 (0-8)	0 (0-0)	0 (0-0)	0 (0-0)	0 (0-0)	0 (0-0)	0 (0-0)	0 (0-0)
Age above 18 years, median (q1-q3)	60 (41–71)	63 (53–71)	69 (51–80)	71 (63–79)	68 (54–77)	71 (61–77)	69 (54–78)	69 (59–76)
**Gender (men), n (%)**	49,501 (51)	855 (63)	27,871 (44)	238 (64)	49,348 (49)	726 (64)	28,525 (53)	330 (70)
**Length of stay, median (q1-q3)**	5 (3–9)	32 (19–53)	4 (2–7)	21 (12–35)	4 (2–8)	23 (13–39)	4 (2–7)	21 (12–35)
**In-hospital mortality, %**	2.2	21	2.5	25	3.1	27	2.9	21
**Admission specialty, %**								
*Cardiology*	8	6	10	7	10	11	11	7
*Gastroenterology*	2	4	5	7	5	9	5	7
*Geriatrics*	1	1	4	3	N.A.	N.A.	3	4
*Gynecology*	12	1	12	1	8	0	10	1
*Hematology/oncology*	6	16	N.A.	N.A.	N.A.	N.A.	N.A.	N.A.
*ICU*	2	13	2	18	N.A.	N.A.	2	17
*Internal medicine*	7	6	12	27	15	26	13	30
*Neurology*	13	6	7	5	5	4	7	4
*Orthopedics*	4	2	8	1	4	1	5	1
*Other*	0	0	N.A.	N.A.	1	1	2	1
*Neonatology/Pediatric ICU*	5	16	N.A.	N.A.	N.A.	N.A.	N.A.	N.A.
*Pediatrics*	9	4	11	1	8	1	9	1
*Pulmonary diseases*	4	4	9	12	13	12	9	9
*Surgery*	22	20	16	14	24	28	18	13
*Urology*	3	1	4	4	5	4	5	4

*ICU, intensive care unit; N.A., this specialty was not specified as an admission specialty within the hospital.*

When examining admissions where at least one HOB occurred, the median length of stay for these admissions ranged from 21 to 32 days and in-hospital mortality was 20%–27%. In 8–14% of the admissions, two or more HOBs were identified. Over 15% of admissions with HOB were related to surgery (13-28%), ICU (13%–18%), hematology/oncology (16%) and pediatrics/neonatology (16%) specialties (the latter two specialties observed exclusively in hospital 1) (Table 2). As can be seen in hospital 1, admissions for hematology/oncology specialties had a disproportionately greater rate of HOB compared to the admissions at risk. Hematology patients possibly drive the relatively higher numbers of HOBs in internal medicine wards in hospitals 2, 3 and 4. Finally, 62%–70% of admissions with a HOB were male, where this was approximately half of the patients among the admissions at risk.

**Figure 2.**
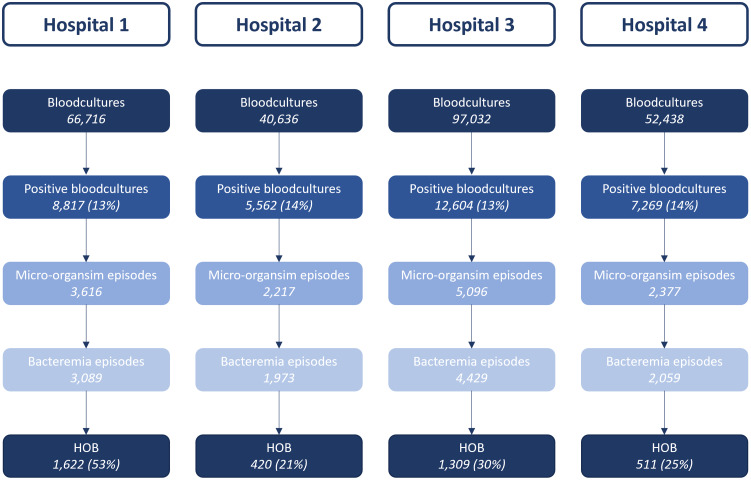
Flowchart from blood cultures to hospital-onset bacteremia. *The percentage presented for HOB indicate the percentage of bacteremia episodes that are hospital-onset*. *For definitions, see* Figure 1.

Figure 2 depicts the classification of blood cultures for each hospital separately. The blood culture rate varied between the hospitals (range 77.7 - 120.4 per 1000 patient days, Table 1), although the percentage positive cultures was comparable between hospitals. Of all bacteremia episodes, 53% was classified as hospital-onset in the tertiary hospital as opposed to 21%–30% in the secondary hospitals.

The hospital-wide HOB rate ranged from 1.0 to 1.9 HOBs per 1000 patient days (Table 3) and was highest in the ICU (8.2 to 12.5 per 1000 patient days). Of note, the study period for hospital 3 and 4 was one year shorter during the pre-COVID period, leading to an increased impact of the COVID period on the overall HOB rates in these hospitals. The HOB rate was very low in neonatal and pediatric departments in hospitals 2, 3 and 4 but was markedly higher in hospital 1, likely due to the dedicated children’s hospital with advanced care facilities. Overall, the median time between admission and all HOBs was 8–13 days (Table 3).

**Table 3.** Description of HOB episodes

	Hospital 1	Hospital 2	Hospital 3	Hospital 4
**Days between admission and HOB, median (q1-q3)**	13 (6–26)	8 (4–14)	9 (4–18)	8 (4–15)
**HOBs, n (rate**^**1**^ **)**				
*Hospital wide*	1622 (1.9)	420 (1.0)	1309 (1.6)	511 (1.1)
*ICU*	464 (8.2)	157 (9.3)	432 (10.8)	175 (12.5)
*Medical wards*	409 (2.3)	111 (0.8)	479 (1.4)	261 (1.0)
*Surgical wards*	309 (1.6)	58 (0.7)	219 (1.0)	41 (0.4)
*Pediatrics/neonatology*	248 (1.7)	4 (0.1)	7 (0.3)	4 (0.2)
*Other wards*	192 (0.7)	90 (0.6)	172 (0.9)	30 (0.4)
**Micro-organism (per HOB), n (%)**				
*Anaerobes*	57 (4)	18 (4)	49 (4)	22 (4)
*CoNS*	316 (19)	50 (12)	185 (14)	103 (20)
*Enterobacterales*	314 (19)	107 (25)	328 (25)	98 (19)
*Enterococci*	320 (20)	51 (12)	202 (15)	70 (14)
*Other*	44 (3)	14 (3)	24 (2)	12 (2)
*Other Gram-negative rods*^*2*^	16 (1)	1 (0)	6 (0)	7 (1)
*Polymicrobial*	251 (15)	59 (14)	209 (16)	94 (18)
*Pseudomonas species*	40 (2)	13 (3)	57 (4)	13 (3)
*Staphylococcus aureus*	153 (9)	61 (15)	168 (13)	55 (11)
*Streptococci*	36 (2)	23 (5)	39 (3)	22 (4)
*Yeasts*	75 (5)	23 (5)	42 (3)	15 (3)
**Composition of polymicrobial HOBs, n (%)**				
*Only pathogens*	123 (49)	25 (42)	107 (51)	33 (35)
*Common commensal and pathogen*	106 (42)	25 (42)	81 (39)	40 (43)
*Only common commensals*	22 (9)	9 (15)	21 (10)	21 (22)

*CoNS, coagulase-negative staphylococci, ICU, intensive care unit.*

^1^ *The HOB rate was calculated as number of HOBs/1000 patient days.*

^2^Other gram negative rods include species from: *Achromobacter, Acinetobacter, Brevundimonas, Burkholderia, Campylobacter, Chryseobacterium, Comamonas, Empedobacter, Pasteurella, Pseudoxanthomonas, Roseomonas, Sphingomonas, Stenotrophomonas, Vibrio.*

**Figure 3.**
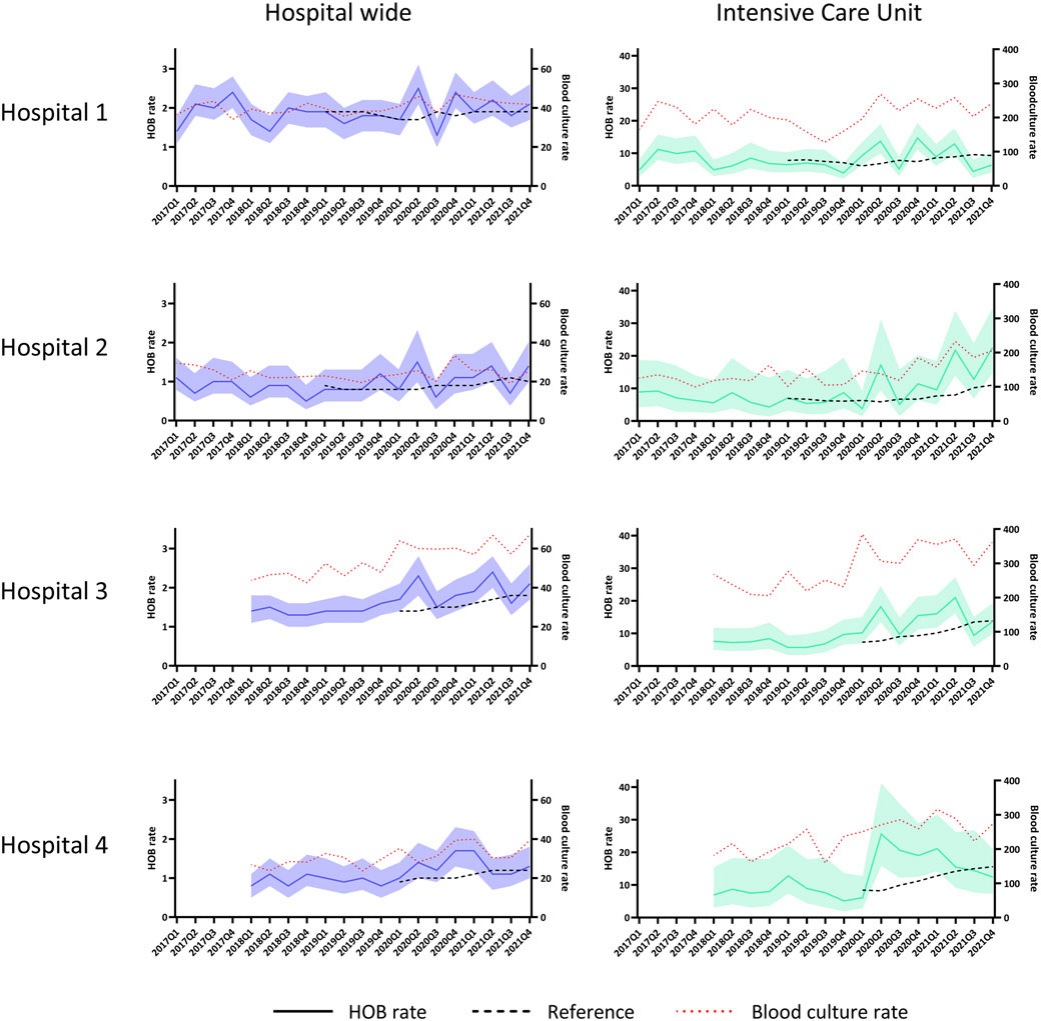
Hospital-onset bacteremia incidences over time. *HOB rates reflected by year and quarter. HOB rate: number of hospital-onset bacteremia episodes per 1000 patient days; reference: mean HOB rate 2 years before the specific timepoint; blood culture rate: number of blood cultures taken per 1000 patient days, reflected at the right y-axis. The band around the HOB rate reflects the 95% confidence interval*.

The HOB rate differs over time in all hospitals, with significantly elevated rates during the COVID-19 pandemic (2020 and 2021) compared to the reference line (Figure 3). The blood culture rate at risk, ie, blood cultures taken 2 or more days after admission, and HOB rate follow similar time trends, at both hospital level and ICU level. HOB rates for medical, surgical and other wards are presented in Appendix 2, Figure S1.

**Figure 4.**
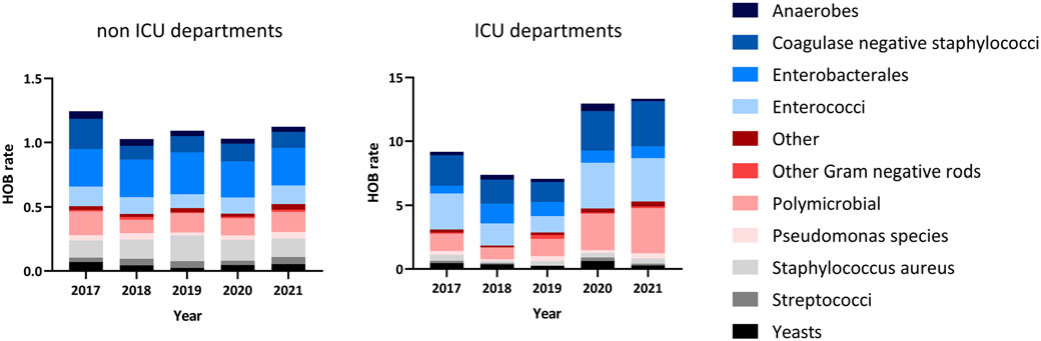
Micro-organism specific HOB rate. *Hospitals were combined in this figure. HOB rate: number of hospital-onset bacteremia’s per 1000 patient days. ICU: intensive care unit*.

HOBs were mostly caused by Enterobacterales, coagulase-negative staphylococci (CoNS), and Enterococci, followed by *Staphylococcus aureus*. In addition, 15%–18% of all HOBs were polymicrobial HOBs (Table 3; micro-organism specific HOB rates are presented in Appendix 3, Table S2). The distribution of micro-organism differs between non-ICU and ICU departments (all hospitals combined); HOB rates for Enterobacterales and *S. aureus* were higher in non-ICU departments, whereas Enterococci and CoNS HOBs were more common in the ICU (Figure 4). The elevated HOB rates during the COVID-19 pandemic are visible in the ICU of all four hospitals, but not in the non-ICU departments. Notably, we observed increased HOB rates specifically for CoNS, Enterococci and polymicrobial HOBs during these years.

### Sensitivity analyses

Using the current definition to confirm common commensals (confirmation culture on day 0, 1 or 2), the vast majority (65%–79%) was confirmed on the same day for the secondary hospitals: 100 (day 0) vs 26 (day 1/2) in hospital 2, 272 vs 147 in hospital 3, and 159 vs 68 in hospital 4. In the tertiary hospital, half of the episodes were confirmed on the same day (283 vs 280) (Appendix 4, Table S3). The current definition identifies the highest number of common commensal HOBs. Limiting the definition to either confirmation cultures taken only on day 0 or confirmation cultures taken only on day 1 or 2 would reduce the number of HOBs from 3862 to 3727 and 3449 respectively, aggregated for all hospitals. As can be expected, this affects mainly HOBs caused by CoNS and polymicrobial HOBs (Appendix 4, Table S4).

Limiting polymicrobial HOB episodes to those when micro-organism episodes start on the same day (as opposed to day 0, 1 or 2), leads to slightly more HOBs identified but less polymicrobial HOBs; however, the impact is limited (16% vs 12% polymicrobial HOBs) (Appendix 4, Table S5). Finally, there were no substantial differences observed in number of HOB episodes when attributed to the sample ward, instead of the attributable ward (Appendix 4, Table S6).

## Discussion

In this retrospective cohort study, we successfully applied fully automated HOB surveillance to data from four Dutch medical centers with different data infrastructures, using the PRAISE consensus definition. Hospital-wide HOB rates varied from 1.0 to 1.9 episodes per 1000 patient days and ICU-level rates varied from of 8.2 to 12.5 per 1000 patient days. These rates are in line with previous studies reporting hospital-wide HOB rates between 0.74 – 2.87 and ICU-level HOB rates between 8.64 and 14.6 per 1000 patient days, albeit these studies applied less standardized and slightly different definitions.^8,11,19^

The median time between admission and HOB varied from 8 to 13 days, consistent with previous findings,^11^ therefore the infections are likely to be truly hospital onset. Furthermore, patients with HOB had a longer length of stay and showed higher mortality, indicating a severely ill patient group. However, we did not study whether this was caused by HOB itself or by underlying risk.

HOBs were predominantly caused by Enterobacterales, CoNS, Enterococci and *S. aureus.* Different pathogens are likely associated with distinct infection sources, for example CoNS are more likely associated with the use of central vascular devices in ICU. Enterobacterales may reflect, amongst others, urinary tract infections and BSI after abdominal surgery. Finally, *S.* aureus is often related to BSI after phlebitis or wounds, eg, surgical site infections. In turn, these sources may be related to the preventability of the infections: sources related to preventable HOBs were often device- or intervention-related.^9,11^ Preventive measures could be targeted to the sources of infection, eg, proper catheter and wound care. Although our study did not assess the sources or preventability of HOB, previous research showed that over 50% of HOBs are potentially preventable.^6,11^ All together, the HOB rate specified by micro-organism could serve as a starting point for local quality improvement.

In addition, monitoring HOB rates over time could highlight areas for local quality improvement. Longitudinal HOB surveillance could detect differences over time at ward-type level (including hospital-specific ward groups) and micro-organism level; increased HOB rates were observed during the COVID-19 pandemic in 2020 and 2021 (Figure 3), in line with the findings of higher catheter-related bloodstream infections in the Netherlands in this period.^20^

Aside from monitoring trends within hospitals and over time, HOB surveillance may in the future also be employed for large-scale surveillance and benchmarking across hospitals. In this setting, ensuring consistency across hospitals is key and grouping wards and/or specialties using eg, the ECDC ward-type classification could be effective to provide meaningful comparisons. However, differences in organizational structure and case-mix between hospitals can complicate benchmarking for specific departments and/or specialties. For example, in three of the four hospitals, the hematology department could not be identified based on ward descriptions as these were integrated within the internal medicine department, although this is a key patient population with increased HOB rates. For benchmarking to be useful, risk-adjustment should be performed to adjust the HOB rate for differences between hospitals. The study of Yu. *et al*^7^ studied the predictors of HOB, and could serve as a starting point for future research in the European situation.

The sensitivity analysis showed that most common commensal episodes are confirmed in separate blood culture sets drawn on the same day. Therefore, a considerable number of HOBs will be missed if day 0 is not part of the confirmation criteria. Importantly, all participating hospitals have protocols specifying that two sets of blood cultures should be drawn when a bloodstream infection is suspected. Variations in blood culture protocols between hospitals, and even between different wards within a hospital, such as pediatric ward or ICUs, could influence the impact of these criteria on the number of HOBs. Future studies should assess these assumptions regarding commensal confirmation in these settings and the clinical relevance of common commensal HOBs.

In our experience, valid data extraction and well-structured data infrastructures are essential for applying an automated HOB surveillance system. The source data required by the algorithm was documented in multiple locations and formats within the EHR and/or LIMS, and data infrastructures may have changed over time or across hospitals. Also, when applying the HOB algorithm, an in-depth understanding of the data sources and clinical microbiology is paramount. In our study, for example, we applied the algorithm to the micro-organism name as stored in the LIMS. For species that cannot be distinguished consistently by MALDI-TOF (eg, species within the *Enterobacter cloacae* complex) this could lead to incorrect identification of new micro-organism events as the copy strains are assigned a different species name. In reporting to the EHR, such inconsistencies are frequently addressed by reporting the species as *Enterobacter cloacae* complex. In theory, both reports could be used as source data for the HOB algorithm and the algorithm should be adapted accordingly. Furthermore, hospital 3 and 4 changed EHR systems in 2017, making data extraction prior to this period impossible. Lastly, data structures differed between hospitals, necessitating data preparation and careful checking to comply with the MDS structure. The coordinating center addressed these challenges successfully, but hospitals will need to handle them individually in future implementations.

As we applied the HOB surveillance system in a research setting, the next step is to implement this system in hospitals for use in daily practice. Our experiences are helpful to design a system that fits the local situation, while using a consensus definition to ensure comparability between hospitals. In addition, implementation should include ongoing maintenance as described by the PRAISE network.^3^ To evaluate this surveillance system once implemented, the existing guidelines for evaluating surveillance systems could be used.^21^ This local initiative and our experiences could inform (inter)national surveillance initiatives. In general, HOB is an objective surveillance target suitable for in a fully automated surveillance, and could monitor trends both within hospitals and at a (inter)national level.

## Strengths and limitations

This study was performed in four hospitals, using hospital-wide data; this is a strength of the study. Furthermore, we used a definition that was recently published and suitable for a large variety of centers, both nationally and internationally. In addition, the results of the sensitivity analysis provide more insight into this consensus definition and its assumptions.

However, this study also has some limitations. Although this was a multicenter study, we only included Dutch hospitals for feasibility reasons, which may limit the generalizability. Moreover, in line with the PRAISE consensus definition, we did not take into account whether a patient was readmitted shortly after discharge or transferred from a different hospital, potentially leading to misclassification of HOBs. Furthermore, although we use the criteria from the PRAISE consensus definition to classify common commensals, the clinical relevance of HOB requires further study, in particular for patients without central vascular devices.

## Conclusion

Applying an automated surveillance system using a consensus definition in multiple centers with different infrastructures is feasible, with the ability to detect differences over time at ward-type and micro-organism-level. Additionally, we showed the impact of the assumptions made in the consensus definition of PRAISE. The results and their visualization could serve as a starting point for infection prevention practices. We recommend future studies to investigate preventability and sources of HOB, and to develop risk-adjustment methods to facilitate benchmarking.

## Supporting information

Brekelmans et al. supplementary material Brekelmans et al. supplementary material

## Supplementary material

The supplementary material for this article can be found at https://doi.org/10.1017/ice.2025.29

## Thank you notes

N.A.
