# Supplementary material for: Automated surveillance of hospital-onset bacteremia and fungemia: feasibility and epidemiological results from a Dutch multicenter study
Source: Infect Control Hosp Epidemiol. 2025 Feb 26;46(5):472–80. doi: 10.1017/ice.2025.29 (PMC12034453; doi:10.1017/ice.2025.29)
Supplement: Brekelmans et al. supplementary material [file S0899823X25000297sup001.docx]

**Appendices**

**Appendix 1: Data specifications**

**Table S1: Data specifications for data collection**

| Type of data | Variable |
| --- | --- |
| Patient-related data | Pseudonymized patientID [1] |
|  | Age [1] |
|  | Gender [1] |
| Blood culture-related data | PatientID [1] |
|  | Sample date [0..*] |
|  | SampleID [0..*] |
|  | Result [0..*] |
|  | Micro-organism [0..*] |
| Admission-related data | PatientID [1] |
|  | AdmissionID [1] |
|  | Admission date hospital [1] |
|  | Discharge date hospital [1] |
|  | Admission date ward [1..*] |
|  | Discharge date ward [1..*] |
|  | WardID [1..*] |
|  | Discharge destination [1] |
|  | Admission specialty [1] |

[1]: 1 result per admission

[0..*]: 0 to multiple results per admission

[1..*]: 1 to multiple results per admission

*In all hospitals, data were collected in two or three datasets (patient- and admission-related data were combined in some centers). Data were converted to the minimal dataset of the PRAISE consensus definition. Merging was performed in the coordinating center, based on patientID and calendar dates.*

**Appendix 2: Incidences of hospital-onset bacteremia over time**

**Figure S1:** Incidences of hospital-onset bacteremia over time


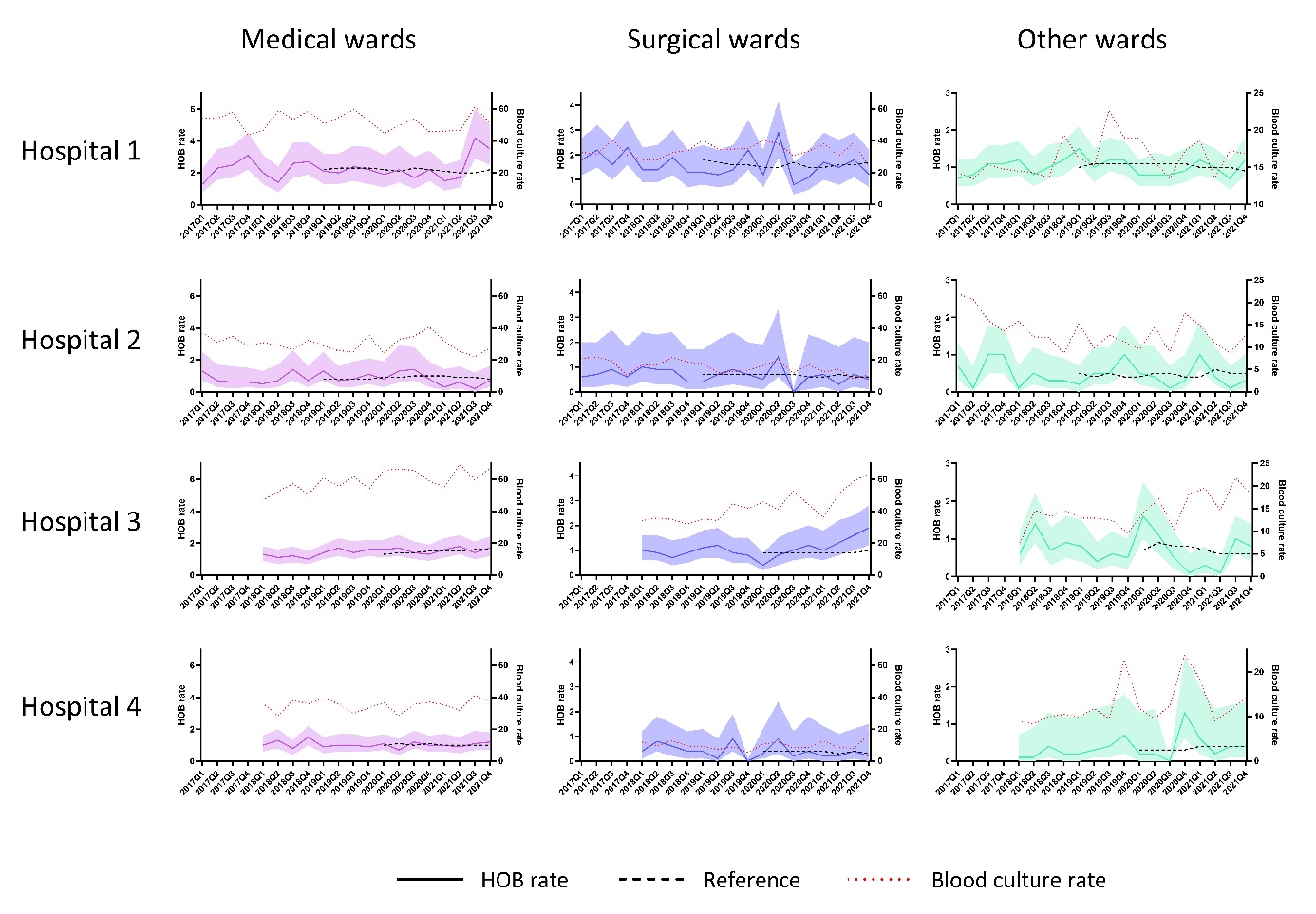


*HOB rates reflected by year and quarter. HOB rate: number of hospital onset bacteremia episodes per 1000 admission days; reference: mean HOB rate 2 years before the specific timepoint; blood culture rate: number of blood cultures taken per 1000 patient days, reflected at the right y-axis. The band around the HOB rate reflects the 95% confidence interval.*

**Appendix 3: HOB rates per microorganism group**

**Table S2: HOB rates per microorganism group**

| Microorganism group, HOB rate | Hospital 1 | Hospital 2 | Hospital 3 | Hospital 4 |
| --- | --- | --- | --- | --- |
| *Anaerobes* | 0.066 | 0.041 | 0.061 | 0.047 |
| *CoNS* | 0.367 | 0.113 | 0.231 | 0.221 |
| *Enterobacterales* | 0.365 | 0.242 | 0.409 | 0.211 |
| *Enterococci* | 0.372 | 0.116 | 0.252 | 0.150 |
| *Other* | 0.051 | 0.032 | 0.030 | 0.026 |
| *Other Gram-negative rods^1^* | 0.019 | 0.002 | 0.007 | 0.015 |
| *Polymicrobial* | 0.292 | 0.134 | 0.261 | 0.202 |
| *Pseudomonas species* | 0.046 | 0.029 | 0.071 | 0.028 |
| *Staphylococcus aureus* | 0.178 | 0.138 | 0.210 | 0.118 |
| *Streptococci* | 0.042 | 0.138 | 0.210 | 0.118 |
| *Yeasts* | 0.087 | 0.052 | 0.052 | 0.032 |

The HOB-rate is calculated as number of HOBs per 1000 patient days.

**Appendix 4: Sensitivity analysis**

**Table S3.** Timing of confirmation common commensal bacteremia episodes

|  | Common commensal confirmed on | | |
| --- | --- | --- | --- |
|  | Day 0 (n) | Day 0 and day 1 or 2 (n) | Day 1 or 2 (n) |
| Hospital 1  Hospital wide  ICU  Non-ICU | 213  61  152 | 70  21  49 | 210  89  121 |
| Hospital 2  Hospital wide  ICU  Non-ICU | 81  49  32 | 19  13  6 | 7  5  2 |
| Hospital 3  Hospital wide  ICU  Non-ICU | 203  105  98 | 69  45  24 | 78  52  26 |
| Hospital 4  Hospital wide  ICU  Non-ICU | 126  88  38 | 32  17  15 | 36  28  8 |

The table presents at what timing the common commensal episodes are confirmed (day 0, day 0 AND day 1 or 2, or day 1 or 2 only). Results are presented for hospital wide numbers, and specifically for ICU and non-ICU departments, for each hospital separately.

ICU: intensive care unit

**Table S4: Micro-organism distribution at the different scenarios for confirmation of common commensals**

| Micro-organism group | Day 0, n | Day 1 or 2, n | Day 0, 1 or 2, n |
| --- | --- | --- | --- |
| *Anaerobes* | 145 | 145 | 146 |
| *CoNS* | 545 | 341 | 654 |
| *Enterobacterales* | 849 | 862 | 847 |
| *Enterococci* | 672 | 704 | 643 |
| *Other* | 91 | 86 | 94 |
| *Other Gram-negative rods* | 30 | 30 | 30 |
| *Polymicrobial* | 557 | 466 | 613 |
| *Pseudomonas species* | 123 | 124 | 123 |
| *Staphylococcus aureus* | 440 | 443 | 437 |
| *Streptococci* | 119 | 87 | 120 |
| *Yeasts* | 156 | 161 | 155 |
| Total | 3727 | 3449 | 3862 |

*Number of HOBs identified using the different assumptions for confirming common commensal episodes.*

*CoNS: coagulase negative staphylococci*

**Table S5: Sensitivity analysis for polymicrobial episodes**

|  | Day 0,1 and 2 (polymicrobial/total) (%) | Day 0 (polymicrobial / total) (%) |
| --- | --- | --- |
| Hospital 1 | 251/1622 (15) | 195/1713 (11) |
| Hospital 2 | 59/420 (14) | 54/435 (12) |
| Hospital 3 | 209/1309 (16) | 162/1392 (12) |
| Hospital 4 | 94/511 (18) | 79/538 (15) |
| Total | 613/3863 (16) | 490/4078 (12) |

*Number of polymicrobial HOBs / total number of HOBs when classifying episodes starting on day 0,1,2 as polymicrobial (main assumption) or only on day 0.*

**Table S6: Sensitivity analysis for attributable ward**

|  | **Attributable ward** | **Sample ward** |
| --- | --- | --- |
| Hospital 1 |  |  |
| HOBs (rate)  ICU  Medical wards  Surgical wards  Pediatrics/neonatology  Other wards | 464 (8.2)  409 (2.3)  309 (1.6)  248 (1.7)  192 (0.7) | 473 (8.4)  403 (2.2)  316 (1.7)  250 (1.7)  189 (0.7) |
| Hospital 2 |  |  |
| HOBs (rate)  ICU  Medical wards  Surgical wards  Pediatrics/neonatology  Other wards | 157 (9.3)  111 (0.8)  58 (0.7)  4 (0.1)  90 (0.6) | 145 (8.6)  122 (0.9)  52 (0.6)  4 (0.1)  97 (0.6) |
| Hospital 3 |  |  |
| HOBs (rate)  ICU  Medical wards  Surgical wards  Pediatrics/neonatology  Other wards | 432 (10.8)  479 (1.4)  219 (1.0)  7 (0.3)  172 (0.9) | 455 (11.4)  494 (1.5)  223 (1.1)  7 (0.3)  138 (0.7) |
| Hospital 4 |  |  |
| HOBs (rate)  ICU  Medical wards  Surgical wards  Pediatrics/neonatology  Other wards | 175 (12.5)  261 (1.0)  41 (0.4)  4 (0.2)  30 (0.4) | 174 (12.5)  264 (1.0)  41 (0.4)  4 (0.2)  26 (0.4) |

*The number of HOBs per ward type specialty are presented, for attributable ward (main assumption) and sample ward.*
